# The tract terminations in the temporal lobe: Their location and associated functions

**DOI:** 10.1016/j.cortex.2016.03.013

**Published:** 2017-12

**Authors:** Claude J. Bajada, Hamied A. Haroon, Hojjatollah Azadbakht, Geoff J.M. Parker, Matthew A. Lambon Ralph, Lauren L. Cloutman

**Affiliations:** a Neuroscience and Aphasia Research Unit (NARU), School of Psychological Sciences, The University of Manchester, UK; b Manchester Academic Health Science Centre, Manchester, UK; c Centre for Imaging Sciences, Institute of Population Health, The University of Manchester, Manchester, UK

**Keywords:** White matter, Tractography, Temporal lobe, Diffusion MRI, Brain mapping

## Abstract

Temporal lobe networks are associated with multiple cognitive domains. Despite an upsurge of interest in connectional neuroanatomy, the terminations of the main fibre tracts in the human brain are yet to be mapped. This information is essential given that neurological, neuroanatomical and computational accounts expect neural functions to be strongly shaped by the pattern of white-matter connections. This paper uses a probabilistic tractography approach to identify the main cortical areas that contribute to the major temporal lobe tracts. In order to associate the tract terminations to known functional domains of the temporal lobe, eight automated meta-analyses were performed using the Neurosynth database. Overlaps between the functional regions highlighted by the meta-analyses and the termination maps were identified in order to investigate the functional importance of the tracts of the temporal lobe. The termination maps are made available in the Supplementary Materials of this article for use by researchers in the field.

## 1 Introduction

The temporal lobe has been implicated in a multitude of cognitive domains, including audition (Kaas & Hackett, 1999), vision (Goodale and Milner, 1992, Grill-Spector and Malach, 2004), language (Cloutman, 2013, Price, 2010), memory (Scoville & Milner, 1957) and semantic processing (Lambon Ralph, 2014). The successful execution of these higher cognitive functions is not carried out by the temporal lobe alone, but requires a complex interaction with other widely distributed brain regions. This interaction is underpinned by the white matter – the brain's information ‘super-highway’ – which consists of bundles of neural axons that carry information over long distances from one area of cortical grey matter to another (Curran, 1909, Martino et al., 2010a, Sarubbo et al., 2013). While information processing occurs in the cortical grey matter, the white matter connections govern the nature and flow of information to and from this grey matter. Indeed, neuroanatomical, neurological and computational accounts have all emphasised that the form and pattern of the white matter connectivity will exert a strong influence over the nature of the neural computation in each area (Binney et al., 2010, Brodmann, 1909, Mesulam, 1998, Plaut, 2002). These hypotheses have been supported by experimental surgical reorganisation of the major auditory and visual pathways in animals (Roe et al., 1992, Sharma et al., 2000, Sur et al., 1988). Accordingly, in order to understand the roles played in higher cognition by different cortical regions, it is important to understand its underlying structural connectivity in detail.

Previous work on the connectivity of the temporal lobe has identified six major association fibre tracts [uncinate fasciculus (UF), inferior longitudinal fasciculus (ILF), inferior fronto-occipital fasciculus (IFOF), middle longitudinal fasciculus (MdLF), arcuate fasciculus (AF) and cingulum] and two main commissural fibres [the corpus callosum and anterior commissure (AC)] that lie within the temporal lobe (Catani et al., 2003, Catani et al., 2005, Catani and Mesulam, 2008, Curran, 1909, Davis, 1921, Déjerine and Dejerine-Klumpke, 1895, Forkel et al., 2014, Makris et al., 2009, Tusa and Ungerleider, 1985). The extreme capsule (EmC) has also been suggested to constitute an important tract within the ventral language system (Makris and Pandya, 2009, Schmahmann and Pandya, 2007, Schmahmann and Pandya, 2009). However, this is with some controversy, and there is a growing consensus that the capsule represents a region within the brain where several tracts converge, and is not a specific tract itself (Bajada et al., 2015, Duffau et al., 2013). As such, the temporal connections of the EmC can either be considered to be part of the IFOF (Duffau et al., 2013), part of an adjacent temporo-frontal fasciculus (Petrides, 2013) or part of the UF. Due to dissection, tracer and magnetic resonance imaging (MRI) studies, much is known about the trajectory of these temporal fibre bundles (Martino et al., 2010b, Martino et al., 2011, Menjot de Champfleur et al., 2013), but less is known about where these tracts originate and terminate.

Dissection methods (e.g., Curran, 1909, Maldonado et al., 2013, Martino et al., 2010a, Martino et al., 2010b, Sarubbo et al., 2013), are generally poorly suited to determining tract terminations for several reasons. First and foremost, the destructive nature of the dissection method poses the greatest challenge in determining the cortical termination points of a given tract: one of the first steps in the process is cortical removal in order to expose the tract under examination, and thus the termination regions of interest are largely destroyed by this method. In addition, in order to perform a dissection, strong prior information regarding tract location is required and many subjective decisions must be made throughout the procedure before reaching the termination of a tract (Curran, 1909). Additionally, the limited number of samples available for dissection makes it difficult to comment with confidence on the variability of tract termination patterns across a population. Finally, only a small number of tracts can be targeted within a single brain and replication on the same brain is impossible.

Tracer studies, in contrast, are able to provide very precise data regarding the terminations of a given tract. In this method, a visualisable tracer agent (such as horseradish peroxidase) is injected into a cortical region of interest, enabling the afferent and efferent neural pathways connecting this region to other areas of the brain to be delineated (Hackett, Stepniewska, & Kaas, 1998). Like anatomical dissection, however, the results of these studies are guided by strong anatomical priors that dictate where to inject the tracer. Also, as the animal is sacrificed in order to examine the path of the tracer, replication in the same brain is impossible. In addition, the tracer method allows only a limited number of injection sites, meaning that only a proportion of a cortical region of interest can be covered in a single specimen. Most importantly, due to the invasive nature of the technique, all data are from non-human primates and other animals, requiring extrapolation to human anatomy which is not precise.

Diffusion MRI tractography is a method that allows one to examine multiple white matter tracts in the same brain. Since the data are not destroyed in the analysis process, the examination can be replicated within the same brain. In this method, the trajectory of fibre bundles is inferred by the orientational preference of movement of water molecules within the brain (Parker, Haroon, & Wheeler-Kingshott, 2003). Although recent years have seen great technical advancements in the analysis of white matter bundles using diffusion imaging (Basser, 1998, Behrens et al., 2003, Makris et al., 1997, Mangin et al., 2013, Mori et al., 1999, Parker et al., 2003), to date, the majority of tractography studies have focused on delineating the course of these white matter pathways and not their specific cortical terminations within the cortex. As such, while much is known regarding the relative trajectories of the fibre bundles within the temporal lobe, precisely where they begin or end remains unclear.

In the current paper, we use probabilistic tractography (Parker et al., 2003) to map the tract terminations of key fibre bundles within the temporal lobe. In order to delineate the cortical terminations of these tracts rather than their trajectories, tractography was first performed from the entire temporal lobe and the resulting output used to identify specific tracts of interest. Regions of the cortex that contributed to the tractographic output for a given tract were then identified, linking the tract to its terminations within the temporal cortex. This approach allowed for the generation of tract termination maps that provide information regarding the probability of a voxel within the temporal lobe having connections via a particular tract of interest. The resultant termination maps are provided in the Supplementary Materials for use by the research community. In addition to delineating the areas of the temporal cortex connected via these key white matter tracts, the potential functional roles of these temporal pathways were also explored. To that end, eight automated meta-analyses (episodic memory, hearing, speech perception, phonological processing, speech production, semantic cognition, social cognition and vision) were carried out using Neurosynth (Yarkoni, Poldrack, Nichols, Van Essen, & Wager, 2011). The overlap of the meta-analyses maps with the tract termination maps were examined in order to probe which tract terminations were associated with which specific cognitive function(s).

## 2 Methods

### 2.1 Image acquisition

Structural (T_1_-and T_2_-weighted) and diffusion-weighted MR imaging datasets were acquired in 24 healthy participants (mean age 25.9 years, range 19–47 years; 11 females). All participants were right handed, as determined by the Edinburgh Handedness Inventory (Oldfield, 1971). This study was approved by the local ethics committee and all participants gave informed consent. The images were acquired on a 3 T Philips Achieva scanner (Philips Healthcare, Best, The Netherlands), using an 8 element SENSE head coil. Diffusion-weighted images were acquired with a pulsed gradient spin echo echo-planar sequence with echo time (TE) = 59 msec, repetition time (TR) ≈ 11,884 msec [cardiac gated using a peripheral pulse monitor on the participant's index finger (n = 21), or using electrocardiography (n = 3)], maximum diffusion sensitisation gradient amplitude *G*_max_ = 62 mT/m, half scan factor = .679, 112 × 112 image matrix reconstructed to 128 × 128 using zero padding, reconstructed in-plane voxel resolution 1.875 × 1.875 mm^2^, slice thickness 2.1 mm, 60 contiguous slices, 61 non-collinear diffusion sensitization directions at *b* = 1200 sec/mm^2^ (*Δ* = 29.8 msec, *δ* = 13.1 msec), 1 at *b* = 0 sec/mm^2^, SENSE acceleration factor = 2.5. In order to correct susceptibility-related image distortions, two volumes were obtained for each diffusion gradient direction with inversed phase encode directions, with distortion correction carried out using the method described by Embleton, Haroon, Morris, Lambon Ralph, and Parker (2010). The distortion corrected images were visually inspected and compared with a co-localized T_2_-weighted turbo spin echo scan (in-plane voxel resolution of .94 × .94 mm^2^, slice thickness 2.1 mm) to provide a qualitative indication of distortion correction accuracy. A high resolution structural T_1_-weighted 3D turbo field echo inversion recovery scan (TR ≈ 2000 msec, TE = 3.9 msec, TI = 1150 msec, flip angle 8°, 256 × 205 image matrix reconstructed to 256 × 256, reconstructed in-plane voxel resolution .938 × .938 mm^2^, slice thickness .9 mm, 160 slices, SENSE factor = 2.5), was acquired in order to obtain high accuracy anatomical data on individual subjects.

### 2.2 Tracking

A temporal lobe seed region was first created for tracking which encompassed all voxels within the temporal cortex at the boundary between the grey matter and the white matter. To do this, for each participant, the structural T_1_-weighted image was first skull- and scalp-stripped using FSL's brain extraction tool (BET) (Smith, 2002), and co-registered to the distortion-corrected *b* = 0 image using a linear affine transformation (FLIRT) (Jenkinson et al., 2002, Jenkinson and Smith, 2001). The co-registered T_1_-weighted image was segmented using FSL FAST (Zhang, Brady, & Smith, 2001) to obtain a white matter mask. Using an in-house MATLAB script (The MathWorks, 2012), all of the voxels on the external surface of the white matter mask were extracted in order to create a grey–white interface (GWI) image for the whole brain. Only GWI voxels that overlapped with a temporal lobe mask were kept. The temporal mask was defined in Montreal Neurological Institute (MNI) space according to the MNI structural atlas within FSL (Collins et al., 1995, Mazziotta et al., 2001), and transferred into each participant's native diffusion space using FLIRT affine transformation (Jenkinson et al., 2002, Jenkinson and Smith, 2001) and manually cleaned to ensure that no voxels bled into the frontal lobe. All the voxels outside of the white matter formed part of an exclusion mask for the tracking procedure, in order to avoid anatomically anomalous tracking outputs that ‘jump’ sulcal boundaries and gyri due to cortex-cerebrospinal fluid (CSF) partial volume effects and to avoid intracortical path propagation.

Unconstrained probabilistic tractography was performed in diffusion space from every voxel in the temporal GWI using the probabilistic index of connectivity (PICo) algorithm (Parker and Alexander, 2005, Parker et al., 2003), which sampled the voxel-wise diffusion probability distribution functions (PDFs) generated via the constrained spherical deconvolution (Tournier, Calamante, & Connelly, 2007) and model-based residual bootstrapping method (Haroon et al., 2009a, Haroon et al., 2009b, Jeurissen et al., 2011). 10,000 streamlines were propagated from each seed voxel, with the step size for streamline propagation set to .5 mm. The default stopping criteria were maintained (streamlines were set to stop if they hit the termination mask, if the path length of the streamline was greater than 500 mm, or if the curvature of the streamline over the scale of a voxel was greater than 180°) as per previous works (Binney et al., 2012, Cloutman et al., 2012, Cloutman et al., 2013, Parker et al., 2005) and in line with finding from Azadbakht et al. (2015) showing that curvature constraints and distance correction have little to no effect on the accuracy of tracking.

**Fig. 1:**
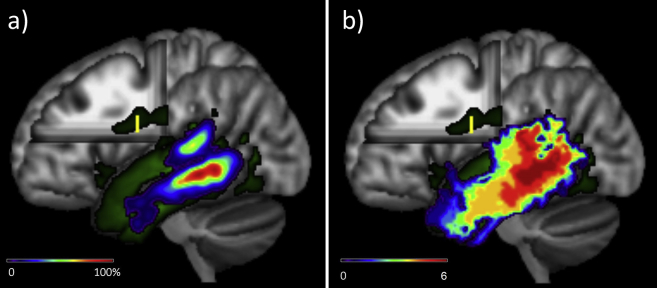
The group-averaged global connectivity profile (green) for the left temporal lobe showing an ROI placed in the main body of the arcuate fasciculus and the resultant termination maps: a) the raw probability map b) the statistical comparison map.

For each individual seed voxel within the temporal lobe GWI (approx. 3000 per individual), the number of streamlines originating from the seed which reached a given voxel in the brain was recorded, generating a connectivity profile for each temporal GWI seed voxel. These connectivity profiles were then added together to create a global temporal lobe connectivity profile for each participant. The individual participant global connectivity profiles were then non-linearly transformed into MNI space using a deformation matrix obtained from the normalisation of the structural T_1_-weighted image to the MNI 152 2 mm template (first by applying FSL FLIRT using an affine transformation and subsequently a non-linear FNIRT) (Andersson, Jenkinson, & Smith, 2010), and averaged across individuals to create a group temporal lobe connectivity profile (see Fig. 1).

### 2.3 Tract region of interest (ROI) placement

Five tracts that pass through or terminate in the temporal lobe were identified on the group connectivity profile: the AF, the AC, the splenium of the corpus callosum, the cingulum, the MdLF. Two more tract complexes were also identified. These differed from the ‘pure’ tracts as they involved several overlapping tracts coursing through the same area. The first tract complex was comprised of those fibre bundles that exited the temporal lobe into the occipital lobe (the occipital fibre complex, Occ), including the ILF and those branches of the IFOF that terminate in the temporal lobe or, depending on the nomenclature used, a temporo-frontal fasciculus (Bajada et al., 2015, Duffau et al., 2013, Petrides, 2013). The second tract complex involved fibres that coursed through the EmC, and included the UF and the IFOF/temporo-frontal fasciculus (Bajada et al., 2015, Duffau et al., 2013, Martino et al., 2010b, Petrides, 2013).

A region of interest (ROI) was drawn in the body of each target tract. To ensure consistent placement across participants and to reduce the requirement for in depth *a priori* anatomical knowledge, ROIs were defined on and guided by the group averaged global connectivity profile normalised to MNI space. The ROIs were then transformed into each participant's native space using FSL FNIRT non-linear transformation (trilinear interpolation and binerisation at .2), and visually inspected to ensure that there were no substantial differences in location from group space and individual space. All ROIs were defined in one plane around a centroid coordinate (Table 1) and were one voxel (2 mm isotropic) thick (further details of ROI placement below and see S1 in supplementary text). The positions of each ROI in MNI space were as follows:

**Table 1:** MNI coordinates for the centroid of each ROI.

	Left hemisphere			Right hemisphere		
	x	y	z	x	y	z
AC	−6	0	−7	8	1	−7
Splenium	−4	−36	15	4	−36	16
Cingulum	−12	−48	8	15	−46	8
AF	−33	−2	26	32	0	23
MdLF	−17	−57	43	21	−54	44
EmC	−27	10	−11	29	10	−10
Occ	−30	−72	5	32	−72	6

AC = anterior commissure, AF = arcuate fasciculus, MdLF = middle longitudinal fasciculus, EmC = extreme capsule fibre complex, Occ = occipital fibre complex.

#### 2.3.1 AC

The AC can be observed branching medially from the anterior temporal lobe to decussate just anterior to the fornix. The ROI was defined in the sagittal plane on the ipsilateral side, slightly anterior and lateral to the anterior pillars of the fornix just before the AC decussates to the contralateral hemisphere. The extent of the ROI was guided by the global connectivity profile.

#### 2.3.2 Splenium of the corpus callosum

The most visible part of the corpus callosum as viewed on the global connectivity profile is the splenium, confined to the posterior most end of the corpus callosum. The ROI was defined in the transverse plane and the extent of the ROI was guided by the global connectivity profile which did not bridge anteriorly into the body of the corpus callosum.

#### 2.3.3 Cingulum

On the global connectivity profile, the cingulum can be clearly seen starting in the hippocampus and arcing round into the white matter of the cingulate gyrus. The ROI was defined in the transverse plane adjacent to the splenium of the corpus callosum.

#### 2.3.4 AF

The AF can be seen hooking round the posterior extent of the Sylvian fissure to course through the extent of the parietal and frontal lobe. The ROI was defined in the coronal plane just as the AF enters the frontal lobe in the plane of the postcentral gyrus just superior to the insular cortex. The extent was guided by the global connectivity profile.

#### 2.3.5 MdLF

This tract was clearly seen on the global connectivity profile as it branches out of the temporal lobe and courses toward the parietal lobe medial and posterior to the main branch of the AF. The ROI was defined in the transverse plane in an area in the white matter just inferior to the superior parietal lobule (SPL) at the level of the supramarginal gyrus. The extent of the ROI was guided by the global connectivity profile. Traditionally, the MdLF has been argued to terminate in the angular gyrus of the inferior parietal lobule (IPL) (Menjot de Champfleur et al., 2013), however there is evidence to indicate that it may actually terminate in the SPL as well as the IPL (Wang et al., 2013).

#### 2.3.6 The EmC fibre complex

The complex is clearly visible on the global connectivity profile as it emerges from the temporal lobe and courses into the ventral frontal cortex via the EmC complex. The ROI was defined in the coronal plane and positioned in the body of the UF as it emerged into the frontal lobe, in the EmC, lateral to the putamen.

#### 2.3.7 The occipital fibre complex

The ROI was defined in the coronal plane and was placed in the occipital lobe at the level of the apex of the posterior horn of the lateral ventricle.

### 2.4 Termination maps

For each tract, a probability-based temporal lobe termination map was generated which was computed by calculating the maximum number of PICo streamlines a particular voxel contributed to a particular tract. For example, if 1000 streamlines from voxel *x* contributed to tract *y*, the intensity of voxel *x* on the termination map would be .1 (1000/10,000). These maps were then non-linearly transformed to MNI space using FNIRT (Andersson et al., 2010), smoothed using a Gaussian kernel with 5 mm FWHM to account for known inconsistency in gyrification patterns across individuals, and then averaged across participants to generate raw unthresholded probability-based temporal lobe termination maps for each tract under examination. The final raw unthresholded maps were rescaled from 0 to 1, hence the maximum point on this map denotes the most probable area that a tract would terminate (given value 1) while the minimum point was the least probable area (given value 0). Consequently, the most probable termination point always had a value of 1 (see Supplementary Materials for the full maps).

Statistical termination maps were also generated, which identified voxels within the temporal GWI which were significantly more likely to be the termination point of a particular tract of interest than other tracts. To do this, a pairwise comparison of all, un-rescaled, termination maps was computed using FSL randomise (thresholded at *p* < .05 familywise error (FWE) corrected within the temporal lobe volume, as well as Bonferroni corrected for multiple comparisons) (Nichols & Holmes, 2002). For example, voxels within the temporal GWI where the AF had a higher probability of termination than other tracts were identified by performing individual tract comparisons between the AF termination map and those of each other tract examined (i.e., AF > AC, AF > Splenium, AF > Cingulum, AF > MdLF, AF > EmC, AF > Occ). The resultant significant voxels from each pairwise comparison map were then summed together. This provided a summary map showing voxels that were consistently more likely to be a specific tract's termination sites than any other fibre tract, on a scale of 0–6, where 0 = greater than no other tract, and 6 = greater than all other tracts (see Fig. 1b for an example of the AF map). It must be noted that at the lowest thresholds these maps may potentially be associated with a degree of error. For example, a comparison may show that the AF is more probable to terminate on voxel X than the EmC, however the voxel may not belong to either tract and still have more streamlines only due to different levels of uncertainty or random error in the probabilistic tractography. As the threshold is raised, the level of evidence for a particular tract is increased until at the highest threshold (6, red in Fig. 1) these maps show voxels where there is most evidence of that tract's termination (i.e., the voxel was significant for that tract on all pairwise comparisons). As such, in the current analyses, the termination maps were thresholded to include only those voxels where a given tract had a greater probability of termination compared with at least half of the other tracts examined (i.e., a scale value of 3).

### 2.5 Meta-analyses

To explore the relationship between the cortical termination areas identified for a given tract and the functioning of that temporal region, meta-analyses across eight cognitive domains known to be associated with the temporal lobe were performed using the automated meta-analysis tool Neurosynth (Yarkoni et al., 2011). The cognitive domains examined included: speech perception (n = 81 studies), speech production (n = 86), hearing (n = 104), episodic memory (n = 270), phonological (n = 310), semantic (n = 844), social (n = 1000) and visual (n = 2549). For each cognitive domain, an individual meta-analysis was performed using the domain name as the search term. Neurosynth produced a z-score map that corresponds to the likelihood that a term of interest had been used in a journal article given the presence of activation (c.f. question 15 Neurosynth FAQs http://www.neurosynth.org/faq/#q15). The reverse inference maps thresholded at .01 (false discovery rate (FDR) corrected) were downloaded.

Regions of interest were created for the temporal termination areas of each tract examined. To do this, the statistical termination map for each tract was thresholded at the level of greatest evidence for the existence of terminations of that tract (the two highest values on the map above a threshold of greater than half the tract). This delineated ROIs for each tract which had the greatest evidence of being the areas of termination for that tract, but it should be noted that this did not exclude the possibility of additional contributions to that area from other tracts.

The termination map for the Occ complex region revealed extensive areas throughout the temporal lobe. In order to explore potential differences along the extent of these terminations, a secondary analysis was carried out where the ROI for this tract complex, thresholded at the maximum evidence (greater than all 6 tracts), was divided into three sections: an anterior superior ROI (the section of the termination that covered the superior temporal gyrus), an anterior inferior ROI, and a posterior ROI (divided at the plane of *y* = −27). The temporal terminations of the splenium were not examined in the meta-analyses as no voxels reached the level of evidence required (i.e., there was no area of termination that was greater than at least half the other tracts).

In order to understand which tract terminations were associated with which specific cognitive domains, an overlap measure was used to determine the proportion (P) of each tract termination ROI (A) that overlapped with each meta-analysis map (B) ($P=\frac{A\cap B}{A}$). In order to account for the difference in meta-analysis map size, the overlap was also calculated 10,000 times between each tract termination ROI and a random arrangement of grey matter voxels the size of each meta-analysis map. An overlap was only reported if it was 95% more likely than chance (Bonferroni FWE corrected) given a random map of the same size.

## 3 Results

**Fig. 2:**
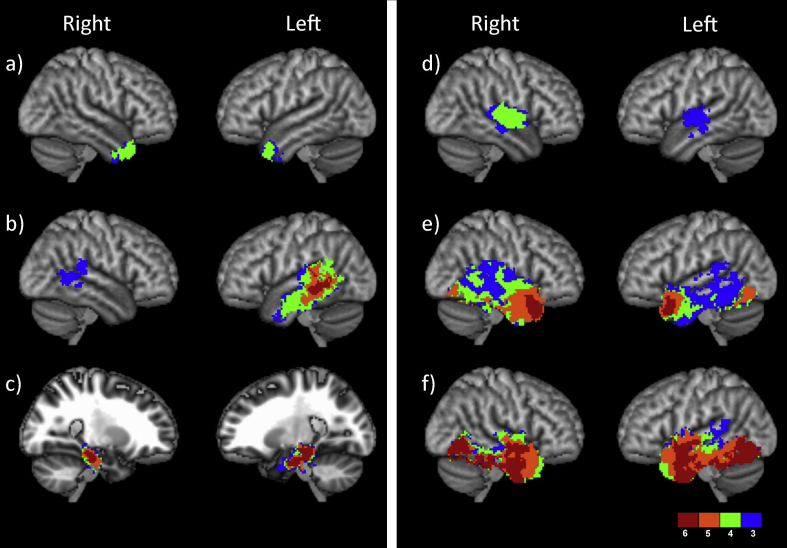
Statistical termination maps for each temporal tract. The colour scale represents the number of tracts had greater evidence for in the pairwise comparisons, thresholded to show voxels that were significant on at least half (three) of the comparisons. a) anterior commissure; b) arcuate fasciculus; c) cingulum; d) middle longitudinal fasciculus; e) the extreme capsule tract complex; f) the occipital tract complex. The splenium is not presented, as it did not demonstrate any voxels above threshold.

The statistical termination maps for each of the tracts are presented in Fig. 2, thresholded to show voxels where a tract demonstrated greater probability of termination compared to at least half the other tracts in the pairwise comparisons (for full, unthresholded maps, see Supplementary Materials). An examination of Fig. 2 reveals that while tracts including the AF, EmC, Occ and cingulum demonstrated strong connections with specific areas within the temporal lobe greater than any of the other temporal tracts (areas in red), both the AC and MdLF were associated with less connective dominance, with termination areas overlapping with other tracts. Indeed, the splenium failed to show any areas within the temporal cortex where it had greater probability of connection in the pairwise comparisons compared with at least half of the other tracts. This could be an indication of a substantial amount of overlap between the splenial terminations and those of the other temporal tracts which may be more prominent within the lobe. Alternatively, this may have resulted from some potential difficulty in tracking along this particular pathway, although it is unclear why this would occur for the splenium specifically.

**Fig. 3:**
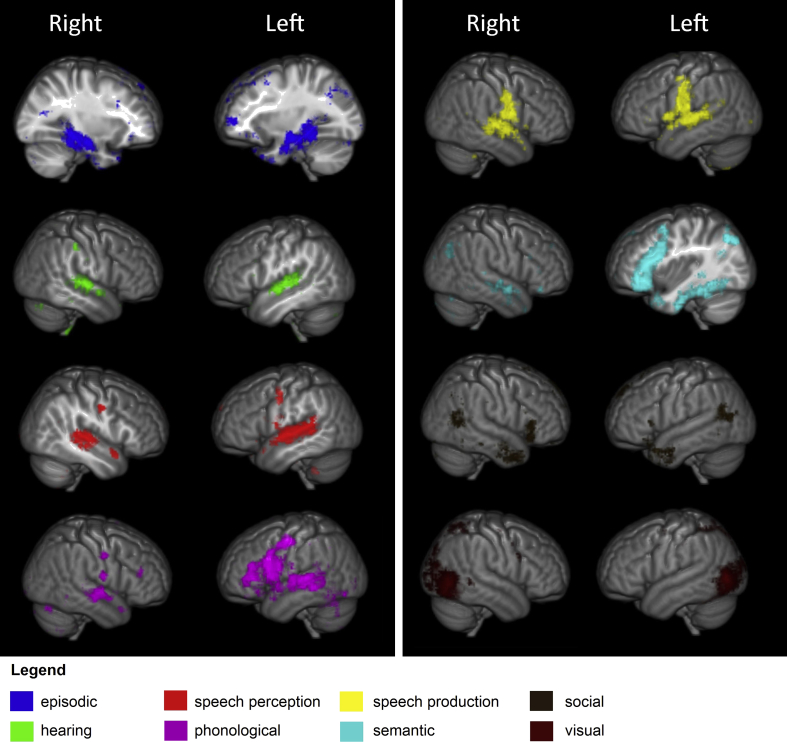
Results of the Neurosynth meta-analyses for the eight cognitive domains examined. Results display the ‘reverse inference maps’ for all regions across the entire brain, thresholded at .01 FDR corrected.

The results of the meta-analyses for each of the cognitive domains explored are depicted in Fig. 3. Within the temporal lobe, cognitive domains relating to speech and hearing tended to be associated with the posterior superior temporal gyrus. Episodic memory was associated with medial structures within the temporal lobe such as the hippocampal formation and parahippocampal gyrus. Vision was exclusively around the junction of the temporal and occipital lobe. The semantic domain was primarily associated with the ventral temporal lobe (primarily in the left hemisphere) while the phonological domain was associated with more dorsal and posterior structures. The social domain was associated with two unconnected areas in the temporal lobe, one at the anterior-most region, and the other at its postero-dorsal extreme. Finally, while some domains, such as semantics and phonology, showed extensive networks both within temporal and with other areas of the brain (particularly frontal), others were associated with more circumscribed areas.

**Fig. 4:**
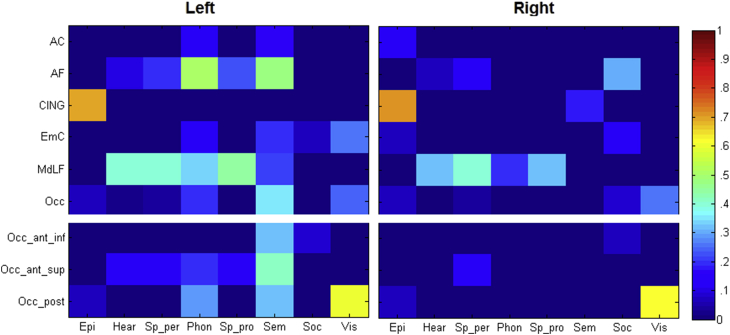
Matrices depicting the proportion of tract termination overlapped by the meta-analysis map of each cognitive domain. Epi = episodic memory, Hear = hearing, Sp_per = speech perception, Phon = phonology, Sp_pro = speech production, Sem = semantics, Soc = social, Vis = visual. Note the upper section is the original overlap with all the tract termination, the lower section sub divides the occipital termination into three components (anterior inferior, anterior superior, posterior).

A matrix showing the proportion of each tract termination map that overlapped with the meta-analyses maps (thresholded at *p* = .05 FWE corrected) is presented in Fig. 4. The termination points of each tract or tract complex and the main functions that were associated with their terminations are described below.

### 3.1 AC

The temporal terminations of the AC are distributed across the temporal pole in both hemispheres (Fig. 2a). There is no extension into the superior temporal gyrus outside of the temporal pole in either hemisphere. Previous studies have suggested that the AC's temporal fibres project from the temporal pole, rostral superior temporal cortex, inferotemporal areas and parahippocampal gyrus (Gloor, 1997), while others argue that it receives contributions from the entire temporal lobe (Reil, 1812, Schmahmann and Pandya, 2009). The current study confirms that the greatest evidence for the AC termination is in the temporal pole, consistent with Gloor's description. Functionally, in the left hemisphere, the phonology (.11) and semantic (.16) meta-analysis maps showed slight overlap with the AC terminations. In the right hemisphere there was some overlap with the episodic memory domain (.14).

### 3.2 Splenium

In the raw maps, the most consistent region of splenium terminations fell in the parahippocampal gyrus that overlapped with the termination of the cingulum, and in the planum temporale just anterior to Heschl's gyrus bilaterally. However, the results of the pairwise comparisons revealed no temporal lobe voxels with splenial terminations which reached the greater than half of other tracts threshold.

### 3.3 Cingulum

The cingulum terminates exclusively on the anterior and posterior division of the parahippocampal gyrus in both hemispheres (Fig. 2c). The current termination results for the cingulum bundle are highly consistent with the classical terminations reported in the literature [cf. Gloor (1997)]. Functionally, the episodic memory domain highly overlapped with the cingulum termination bilaterally (L .69, R .71). In the right hemisphere, the semantic domain also partially overlaps the cingulum termination (.18).

### 3.4 AF

The terminations of the AF are consistently situated in the posterior middle and superior temporal gyrus in both hemispheres (Fig. 2b). These results are consistent with previous studies which have suggested that the AF terminates within the superior temporal gyrus, superior temporal sulcus and middle temporal gyrus (Catani et al., 2005, Cloutman et al., 2013, Schmahmann and Pandya, 2009).

This bundle shows hemispheric asymmetry in both the extent and the level of evidence available for the termination. While there is a core region within the posterior superior temporal gyrus and middle temporal gyrus that shows very strong evidence for AF termination in the left hemisphere, the homologous area in the right hemisphere only shows greater evidence for termination compared to three other fasciculi in the pairwise comparisons. This result is perhaps not surprising given the well documented hemispheric asymmetries of the AF (Catani et al., 2007, Catani and Mesulam, 2008, Parker et al., 2005).

Functionally, the left AF overlapped a number of the cognitive domains examined including phonological (.50), semantic (.48), speech production (.22) and perception (.19) as well as hearing (.10). In the right hemisphere, the AF termination map overlapped with the social domain (.30), as well as the speech perception (.12) and hearing (.06) domains.

### 3.5 MdLF

The MdLF shows moderate evidence of terminating (greater than 3 other tracts and 4 other tracts on the left and right respectively, Fig. 2d) in the superior temporal gyrus bilaterally. The tract terminates on Heschl's gyrus extending to the posterior superior temporal gyrus, anteriorly, into the planum polare and posteriorly into the planum temporale. Previous studies have suggested that the MdLF temporal lobe terminations are along the length of the superior temporal gyrus up to the temporal pole (Makris and Pandya, 2009, Makris et al., 2013, Schmahmann and Pandya, 2009), while this technique suggests a somewhat more restricted termination region.

Functionally, the MdLF termination had similar cognitive associations in both the left and the right hemispheres. They included speech production (L .44, R .32) and perception (L .40, R .40), hearing (L .39, R .38) and phonology (L .34, R .20). In the left hemisphere, the MdLF termination also overlapped with the semantic domain (.22).

### 3.6 EmC complex

The EmC complex has been described a single tract in multiple studies (Makris and Pandya, 2009, Mars et al., 2015, Saur et al., 2008). However, there is a growing consensus that this term describes not an individual tract but the anatomical location between the claustrum and the putamen where several tracts converge including the UF, the IFOF and the temporo-frontal fasciculus (often just called the EmC) (Duffau et al., 2013, Duffau et al., 2014, Martino et al., 2010b, Petrides, 2013). As such, the current study identifies the temporal terminations of all tracts coursing through the EmC as a whole, with interpretations of the individual components of the EmC presented post-hoc.

The anterior most termination of the EmC lies at the superior end of the temporal pole; this is classically the termination site of the UF. Posterior to this, there is an area in both hemispheres along the anterior superior and middle temporal gyri that also has strong evidence for terminations. This is an area that possibly represents the termination of the temporo-frontal fasciculus as described by Petrides (2013). In addition to these more superior terminations, there is also evidence for terminations on the posterior fusiform gyrus, which can be interpreted as consistent with previously described temporal terminations of the IFOF (Duffau et al., 2013).

Functionally, the functional domains that overlap with the EmC termination map in the left hemisphere are visual (.26), semantic (.20), phonology (.12) and social (.06). The overlap in the right hemisphere includes the social meta-analysis map (.12) and episodic memory (.08).

### 3.7 Occipital complex

The Occ tract complex connecting the temporal lobe to the occipital lobe has extensive termination points within the temporal region, with evidence for terminations all along the ventral surface of the temporal lobe bilaterally. This is consistent with terminations of the ILF as well as the occipito-frontal U-fibre projection stream (Catani et al., 2003, Davis, 1921, Tusa and Ungerleider, 1985). Additional terminations within the anterior superior temporal gyrus consistent with ILF are also present. However, this area may be a temporal offshoot of the IFOF.

Functionally, the most extensive overlap with the occipital termination was with the semantic domain (.35) in the left hemisphere, followed by visual (.24), phonology (.20), episodic memory (.08) and speech perception (.04). In the right hemisphere, the visual (.26), episodic (.77) and speech perception (.04) domains overlapped with the termination of the occipital complex of fibres.

In order to perform a more detailed functional overlap analysis, this termination map was further divided into three regions based on prior anatomical knowledge: 1) an anterior inferior region (Occ_ant_inf) covering the anterior sections of the inferior temporal and fusiform gyri, most likely representing the anterior terminations of the ILF; 2) a superior-anterior section covering the anterior middle and superior temporal gyri but excluding the pole (Occ_ant_sup), that potentially also represents a termination site of ILF but which may be a termination point of the tempo-frontal fasciculus as described by Petrides (2013); and 3) a posterior section along the posterior inferior temporal and fusiform gyri (Occ_post), that most likely represents the amalgamation of the posterior terminations of ILF and the posterior temporal branches of the IFOF described by Duffau et al. (2013). The results of the functional overlap analyses revealed that anterior inferior section was associated with the semantic (.32) domain in the left hemisphere, and the social domain bilaterally (L .08, R .07). The anterior superior section was associated with the semantic (.41), phonological (.20), speech production (.14), perception (.13) and hearing (.14) domains in the left hemisphere, but with only speech production (.14) in the right. Finally, the posterior section was associated with vision (L .61, R .61) and episodic memory (L .07, R .07) bilaterally, and additionally with semantics (.33) and phonology (.29) in the left hemisphere.

## 4 Discussion

The current study delineated the cortical terminations of the main tracts and tract complexes within the temporal lobe, and explore their association with key cognitive functions known to be carried out by temporal areas. The termination maps produced revealed differing patterns of connectivity for each of the tracts examined, and provided a framework for understanding the underlying structural architecture which enables the temporal lobe to function in such diverse complex functions as semantics, language production and perception, episodic memory, hearing and vision.

### 4.1 Structure

The temporal lobe tract terminations found in the current study build upon and are primarily consistent with the information and hypotheses derived from previous dissection and tract tracer studies (c.f. Bajada et al., 2015 for a review on the subject). The results also provide some clarifications with regard to debates in the literature. In particular, our results indicate that the ILF terminates at multiple points all the way down the ventral portion of the temporal lobe. This is consistent with both arguments in the controversy over whether the ILF exists as one direct occipito-to-anterior-temporal bundle or whether it is formed from a series of adjacent u-fibres and supports Catani et al. (2003) findings that both a direct and indirect occipitotemporal pathway is present in the human brain. There were also two areas of variation against the exact termination patterns proposed in the literature.

The MdLF was reported by Makris et al. (2013) as terminating along the whole STG. The current results show a distinct peak in the area around Heschl's gyrus and not much of an extension, anteriorly or posteriorly. Secondly, the AC map shows some discrepancy with previous literature. Most of what we know about the AC comes from animal tracer studies and some dissections (Gloor, 1997, Reil, 1812, Schmahmann and Pandya, 2009). The termination map generated for the AC shows a specific peak around the temporopolar cortex which is consistent with Gloor (1997) description of the bundle's termination points. This does not discount the fact that the AC may carry fibres from elsewhere in the temporal lobe but there is not enough evidence in this analysis. It is possible that minor connections are missed due to the thresholding and statistical methods. It could be argued that the method only shows the strongest and most specific connection terminations.

### 4.2 Function

The temporal lobe has been associated with a numerous and diverse range of cognitive domains within the literature. In an attempt to elucidate the complex relationship between cortical function and underlying structural architecture, evidence to support the association between the cortical locus of eight such cognitive domains (as identified via functional imaging), and the termination areas of the main temporal lobe tracts was explored. While the termination maps within the current study do not indicate temporal areas of exclusive cortical termination for any single tract, they do demonstrate those areas with the greatest certainty of connection via a particular tract, enabling the functions associated with each tract to be investigated. Indeed, it is possible, if not highly probable, that cortical regions connected via only one tract are rare, and it is likely that higher-order cognitive functioning relies on input via multiple tracts. In attempting to unravel the functional associations of the main tracts within the temporal lobe, we may further our understanding of their role in these complex functions.

The results from the overlap analyses indicated that episodic memory is primarily associated with the terminations of the cingulum in both hemispheres. This is consistent with studies of both normal and impaired episodic memory which have implicated key medial regions throughout the brain including the hippocampal and parahippocampal gyri in the temporal lobe, as well as the posterior cingulate cortex (Scoville & Milner, 1957). These areas are known to be highly connected via the cingulum which provides the dominant connections to these areas (Catani et al., 2002, Lockhart et al., 2012, Metzler-Baddeley et al., 2011), and damage to the tract has been associated with episodic memory impairments (Bozzali et al., 2012, Lockhart et al., 2012). The additional association between episodic memory and the posterior component of the Occ complex may come from the connections via the IFOF to frontal regions also implicated in the memory domain, or alternatively, may reflect the contribution of other cognitive components required for memory located in temporal areas connected via the Occ complex fibres.

While both episodic memory and vision were found to be bilaterally represented in the tracts associated with these domains, consistent with their known neuroanatomy and functioning, other domains including semantics and those implicated in language, particularly phonology, showed left hemisphere dominance. The finding of left hemisphere dominance in the tract associations with the more linguistic domains is not unexpected and is highly consistent with previous findings (Rosen et al., 2011, Scott et al., 2000). These tracts predominantly involved those which terminated on or around the auditory cortex and the superior posterior temporal regions, including the AF and the MdLF. The left AF particularly was associated with phonology, speech production and perception, much more so than its right hemisphere counterpart, which most likely reflects the domination of this pathway within the dorsal language network (Parker et al., 2005). The strong leftward asymmetry found in its associations with these linguistic domains is highly consistent with studies which have found that it is particularly the AF component connecting posterior temporal regions directly to the inferior frontal lobe (sometimes referred to as the ‘long segment’), which is associated with a leftward dominance in tract volume (Thiebaut de Schotten et al., 2011). The anterior superior component of the Occ was also found to be associated with the more linguistic domains. This may reflect the role of these connections within the ventral language pathway, or reflect the connection to frontal language regions via those Occ tracts which also constitute part of the EmC complex, that is the IFOF and the temporo-occipital fasciculus. These fibres are known to course along the extent of the temporal lobe, connecting posterior temporal and inferior frontal regions important for language (Duffau et al., 2013, Duffau et al., 2014, Martino et al., 2010a).

While the finding of a left hemispheric dominance for the associations with language related functions is unsurprising, the asymmetry found within the semantic domain is unexpected. Consistent with its extensive network throughout the temporal lobe, the semantic domain was found to be associated with almost all of the tracts examined. However, with the exception of the cingulum, which demonstrated a right-sided dominance, the tract associations with the semantic domain were all found within the left hemisphere. A semantic hub has been proposed to be bilaterally represented within the ventral anterior temporal lobe (vATL) (Lambon Ralph et al., 2010, Lambon Ralph et al., 2012, Lambon Ralph et al., 2009, Rice et al., 2015a, Rice et al., 2015b, Schapiro et al., 2013), so this strong association between semantics and the left temporal tracts is somewhat surprising. One possible explanation for this is the fact that many of the studies included in the meta-analysis used in the current study utilised semantic tasks (such as synonym judgement) which involved written words as stimuli. Formal fMRI meta-analyses and targeted MEG comparisons of spoken versus written-word processing have shown that activation laterality is directly influenced by task/modality: most tasks and modalities generate bilateral semantically-related activations with two exceptions – tasks that require spoken output and those that utilise written word stimuli (the most commonly used type of stimulus), for which the activation pattern becomes strongly left lateralised (Marinkovic et al., 2003, Rice et al., 2015b). If correct, then the left-lateralised semantic function obtained in this study is most likely to reflect a biased sampling provided by the studies found in the literature.

An additional finding regarding the tracts associated with the semantic domain is the overlap with the AF tract. Traditionally, semantics has been associated with a ventral network, implicated in time-invariant processing, such as sound-meaning mapping, and has not involved a dorsal pathway contribution (Hickok & Poeppel, 2004). Instead, this dorsal network, and the AF in particular, has been associated with time-variant processes involved in segmentation and sequencing, such as those involved in sound-motor mapping or spatial processing (Weiller, Bormann, Saur, Musso, & Rijntjes, 2011). Examination of the regions of the AF termination map associated with semantics reveals that its overlap with the semantic domain is within the posterior middle temporal gyrus, which is in contrast to its more linguistically associated terminations within the posterior superior temporal region. Posterior MTG is commonly implicated in certain aspects of semantic processing in both neuropsychological and functional neuroimaging studies. There are generally two hypotheses with regard to the function of this region. The first is that pMTG provides an interface between acoustic-phonological processing and lexical-semantic representations (Hickok & Poeppel, 2004). A second hypothesis arises from the observation that executively-demanding semantic tasks also seem to differentially activate pMTG areas (Noonan, Jefferies, Visser, & Lambon Ralph, 2013) and thus this region might provide an interface between temporal lobe semantic representations and frontoparietal executive systems (Binney et al., 2012).

One tract of particular interest given the current findings is the MdLF. The MdLF has traditionally been one of the least studied and consequently most poorly understood of the temporal tracts (Bajada et al., 2015). Indeed, it was only first described as a unique tract within the primate brain as late as 1984 (Seltzer & Pandya, 1984), and was not identified in humans until the late 1990's (Makris, 1999). The current findings add to the growing body of literature regarding the anatomical structure and function of this tract, revealing it to be bilaterally associated with the more language-based domains including phonology, language production and perception, as well as hearing, consistent with previous studies (Saur et al., 2010). The bilateral association with these functions may reflect its connections with Heschl's gyrus and surround areas, with hearing heavily implicated in speech production and perception. The MdLF may constitute part of the ‘repetition-phonological’ network, involved in the acquisition of new and novel words via repetition, and the monitoring and adjustment of speech output (Rauschecker & Scott, 2009). The bilateral nature of the association is consistent with this interpretation, with the repetition network being at its core a sensorimotor integration network. Alternatively, recent computational studies of language have argued that the ventral language pathway is initially underpinned by the MdLF coursing through the temporal lobe (Ueno, Saito, Rogers, & Lambon Ralph, 2011). The current findings suggest that the MdLF may be an important tract implicated in both the dorsal and ventral language pathways.

### 4.3 Methodological considerations

The current study constitutes one of the first to attempt to map the termination locations of the major white matter fibre bundles within the human temporal lobe *in vivo*. To achieve this, probabilistic tractography was used to map the white matter pathways of the temporal lobe, and associate them back to their cortical points of origin. When using any tractographic technique, the resulting findings need to be interpreted with an understanding of the known limitations inherent in the method. Firstly, it is important to acknowledge that tractography is an indirect method of measuring neural tracts. Although it has been validated against gross dissection studies (Kier et al., 2004, Maldonado et al., 2013, Sarubbo et al., 2013), it is known to generate both false positive and false negative tracts. Of particular importance to the current study is the effect of distance on probabilistic tracking, which results from the fact that uncertainty accumulates from voxel to voxel as a function of streamline distance from the seed, and as such streamlines from seed voxels closer to a given region are interest are more likely to reach that region than those from voxels anatomically further away. Various distance correction methods have been proposed, however, these have a tendency to also increase the false positives associated with a distant seed (i.e., they can increase noise) (Azadbakht et al., 2012, Azadbakht et al., 2015). Hence, no distance correction was applied in this study, consistent with the approach used in other studies utilising probabilistic tractography (Binney et al., 2012, Cloutman et al., 2012, Cloutman et al., 2013, Parker et al., 2005). Although, as in all tractography studies, distance is likely to have affected the current results to some extent, there is evidence that it did not have an overall strong effect on the findings. This can be seen in the fact that seed voxels within the posterior temporal lobe were found to have strong evidence for terminations from the EmC [consistent with current views regarding this fibre complex; c.f. Mars et al. (2015)], despite being the furthest points from that particular tract ROI. If distance effects were having a strong impact on the current results, the evidence for these long-range connections would be the most greatly affected, and we may expect to lose these terminations from the termination maps, which did not occur.

Caution must also be taken in the interpretation of the termination maps, produced based on the findings of the pairwise statistical comparisons between tracts. The termination maps produced for a given tract showed voxels within the temporal cortex found to be connected via that tract (or complex), with values indicating which voxels were found to have greater evidence for its existence compared with the other tracts (ranging from 0 = greater than no other tracts, to 6 = greater than all other tracts) based on the pairwise comparisons. The values of the termination maps can hence be considered as a level of evidence that a tract terminates in that particular voxel where one is possible but improbable and six is the most probable. As such, it is important to reiterate that due to tractographical artefacts, statistically, there may be regions found to be significant in a pairwise comparison between tracts which may not reflect the true underlying connectivity of the area, but the specific tracts contrasted. For example, the temporal pole may have be found to be a significant termination point for the AF when contrasted with the cingulum if either or both of these tracts were associated with artefactual tracking from this area, even though *a priori* knowledge would suggest that neither of these tracts terminates in the pole. Given this, regions within the termination maps with low values should be interpreted with caution, and in the current study a threshold was used which limited the results for a given tract to include only those where it demonstrated greater evidence compared to at least half of the other tracts examined, reducing the potential for these significant artefactual terminations.

Within the current study, the meta-analyses used in the functional analyses were obtained using the automated meta-analysis software package Neurosynth, which is freely available online (http://www.neurosynth.org/). A full description of the meta-analysis method used by Neurosynth and its limitations can be found in Yarkoni et al. (2011), but we outline the most salient limitations here. As the procedure used to obtain a Neurosynth meta-analysis is automated, it relies on words or phrases that appear in abstracts as proxies for cognitive functions. While this approach is convenient, it limits the user's ability to define more fine-grained and detailed subcomponents or sub-domains for a given cognitive function. For example, the term “semantic” may be used in the context of lexical semantics, visual semantics etc., and it is important to take this into consideration when interpreting the results of any ‘semantic’ meta-analysis. However, comparisons between Neurosynth meta-analyses of broad terms and more formal meta-analyses of the same cognitive function have been found to produce similar results (c.f. Yarkoni et al., 2011). An additional consideration when using Neurosynth is the fact that the analyses are limited to a database that is accessible to the program. While this limits the scope of the analyses, it does not allow for any user ‘cherry-picking’ as is possible in a less constrained meta-analysis. Finally, it is important to note that the Neurosynth approach utilises an FDR rather than FWE correction for practical computational reasons; hence one must be aware that the maps may contain some locations that are false positives.

## 5 Conclusion

The tract terminations in the temporal lobe have hither to not been comprehensively explored. This study is the first attempt to understand the termination structure of the white matter tracts of the temporal lobe and explore the functional information that they may be responsible for carrying, using non-invasive *in vivo* MR imaging.

## Figures and Tables

**Supplementary Image:**
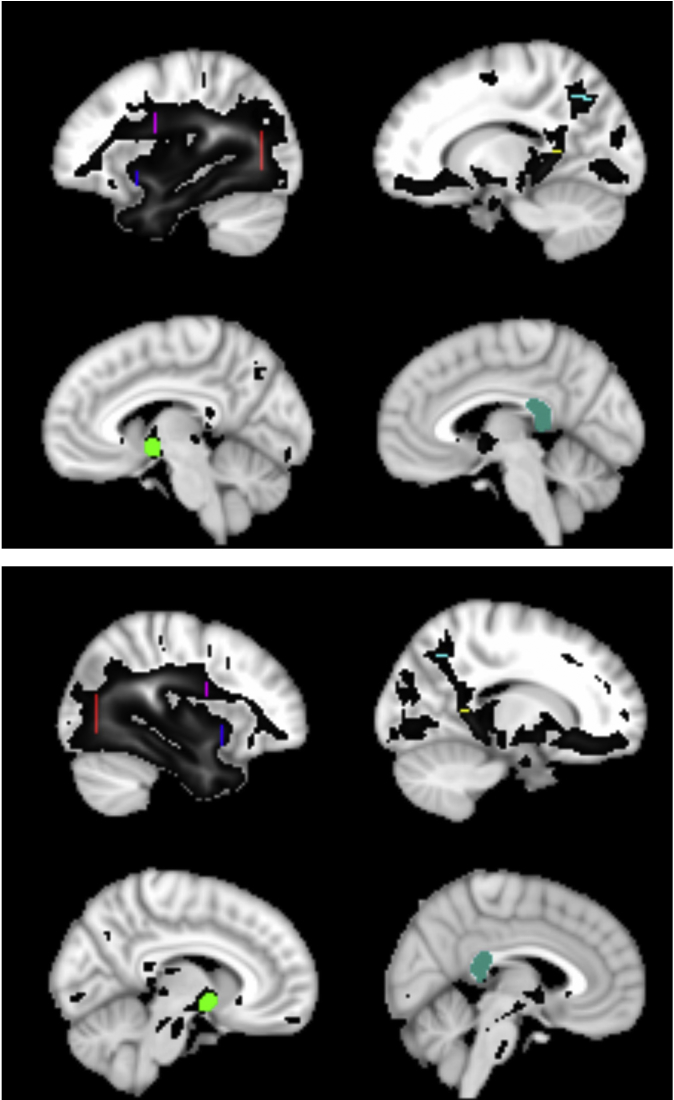
Image of ROI placements to extract the tracts of interest. Blue = Emc; Red = Occipital complex; violet = arcuate; yellow = cingulum; cyan = MdLF; green = AC; turquoise = splenium; black-grey = group connectivity profile used to identify tracts of interest.
